# Comparative evaluation of radiographic and computed tomographic findings in dogs with bilateral medial coronoid disease (MCD) presenting with unilateral forelimb lameness

**DOI:** 10.1371/journal.pone.0282656

**Published:** 2023-04-10

**Authors:** Sophia Seidler, Michaela Rhode, Holger Volk, Oliver Harms

**Affiliations:** 1 Department of Surgery/Orthopaedics, Clinic for Small Animals, University of Veterinary Medicine Hannover Foundation, Hannover, Niedersachsen, Germany; 2 Clinic for Small Animals, Ludwigsburg Ossweil, Baden-Württemberg, Germany; 3 Tierarztpraxis für Kleintierchirurgie, Neuenrade, Nord-Rhein Westfalen, Germany; UTAD: Universidade de Tras-os-Montes e Alto Douro, PORTUGAL

## Abstract

**Objectives:**

The purpose of this study was to compare the radiographic and computed tomographic (CT) findings of dogs with diagnosed bilateral medial coronoid disease, which showed clinically only unilateral lameness of the forelimbs.

**Materials and methods:**

Medical records, including radiographs and CT images of dogs with diagnosed bilateral MCD showing only a unilateral forelimb lameness clinically were reviewed retrospectively. Depending on the gait of each dog we established two groups to investigate their radiographs and CT data comparatively. Group I: affected non-lame limb. Group II: affected lame limb. Several evaluation systems were used to assess which factors are important for clinical decision making and a patient tailored therapeutic plan.

**Results:**

Data from 84 affected elbow joints (42 dogs) diagnosed with MCD by computed tomography were included. Both the radiological and the CT analysis showed that there are significant differences between Groups I and II. Group I had a lower modified International Elbow Working Group Score (IEWG), the values of the Trochlear notch sclerosis were only slightly deviated, and this group showed less often a dislocation of the fragment compared to group II. Furthermore, the size of the fragment (both the median and the mean value) of the forelimbs from group II was almost twice as big as the one from group I. The following sizes of the fragments were calculated (group I versus (vs.) group II)—median: 0.09 cm^2^ vs. 0.16 cm^2^, mean value: 0.112 cm^2^ vs. 0.202 cm^2^. It could be shown that a larger fragment is more likely to dislocate than a smaller one.

**Clinical significance:**

This study provides some evidence towards a better understanding of which diagnostic parameters and findings might be important in clinical decision making. Nevertheless, a “decision tree” for the correct therapy of MCD could not be determined in this study.

## Introduction

Fragmentation and fissuring of the medial coronoid process (MCP), as well as, pathological lesions of the cartilage and the subchondral bone of the medial coronoid process are part of medial coronoid disease (MCD) [1, 2]. MCD alongside three other clinical pictures such as the ununited anconeal process, osteochondritis dissecans (OCD) and elbow incongruity are part of elbow joint dysplasia [2]. Fragmented medial coronoid process is the most common manifestation of elbow dysplasia (ED) and is also a common cause of thoracic limb lameness in medium to large, rapidly growing breeds of dogs with an increased prevalence in males [3–6]. Most cases first present between 5 months and 12 months of age [6–9], and depending on the literature information, there is another group with an incidence of 12% showing first signs of a forelimb lameness at 6 years or older [10]. The clinical picture often shows amongst other pathologies, the following pathological findings in the affected limb: lameness and relief posture, external rotation and pain when there is applied pressure on the medial coronoid [2, 11]. Another interesting finding, described by Moores et al. is that 50% of their study-population of 50 dogs showed abnormalities of the medial coronoid process without any clinical signs of lameness [12]. Abnormalities were described as computed tomographic findings in form of fragmentation, fissures, sclerosis or hypoattenuation as well as an abnormal shape and irregular radial incisures [12]. The aetiology of MCD is undetermined at present, but the literature agrees that it has a multifactorial origin [13]. The implicated factors are genetic dispositions, abnormalities of the underlying subchondral bone and abnormal mechanical loading [2, 13, 14]. Furthermore, other environmental factors such as exercise, nutrition, microtrauma and mineral imbalance cannot be ruled out as to be relevant [2]. Radioulnar incongruity also seems to play an important role [2, 15]. Regardless of a variable sensitivity, radiographic examination is always the first choice for ED screening in practice [16–18]. However, due to both superimposition of the radial head over the medial coronoid process and osteophytes, a correct assessment of the MCP is not always possible [19]. Furthermore, a tight fit between the ulnar trochlear notch and the humeral condyle complicates the correct diagnosis [20]. Often a suspected diagnosis of MCD can only be made based on secondary changes such as osteophytes, blurring of the cranial coronoid contour and sclerosis of the ulnar notch [20, 21]. Further examinations in the form of computer tomographic diagnostics are necessary to confirm the findings, which offer the advantage of more clarity, since images are not superimposed on each other and can be evaluated in different reconstructed views [22–24]. Nevertheless, even by combining the two diagnostic tools radiography and CT, we do not have 100% reliable information about all bone details and of the integrity of articular cartilage [25, 26]. In the literature, many different methods of treating MCD are described, ranging from conservative to minimally invasive and invasive osteotomy or ostectomy [2]. There is no official, unambiguous "protocol" for the therapy of MCD, but all options pursue a return to normal function, ameliorate pain and a slowing of the progression of osteoarthritis [3, 27, 28]. Fragment removal is often recommended, but even if a fragmentation of the medial coronoid process is present, surgical removal of the fragment does not always guarantee a good outcome. This implies that the fragmentation is not necessarily the sole cause of clinical signs [15, 28] and it is still possible, that arthrosis will progress [14, 20, 21, 29, 30]. It is assumed that the removal might result to a load redistribution to either the remaining portion of the medial contact area, or to the lateral elbow contact area including the radial head. Subsequently, this could potentially accelerate cartilage degeneration or cause subchondral pathology at these sites [29]. The incongruence of the radioulnar articular surfaces may lead to an overload of the medial part of the elbow joint, and seems to play a non-negligible role in the clinical symptoms and prognosis [17, 31]. After the removal of the fragment, the incongruence remains and can therefore also influence the outcome. In addition, when comparing different surgical methods regarding fragment removal, the outcomes are very variable [27, 28, 30, 32–36]. In most of these studies no significant improvement of long-term function after fragment removal is observed [27, 28, 30, 33, 34, 37].

It is still ambiguous as to what affects the outcome of dogs with MCD [34]. Many factors such as severity and duration of lameness at the time of presentation, the degree of cartilage damage and osteoarthritis and the type of lesions present in the joint, could all affect outcome and prognosis [34].

The purpose of the current study was to evaluate if there are radiographic and CT findings which can explain the discrepancy of a unilateral forelimb lameness despite a bilateral diagnosed MCD. We hypothesised that there is a significant difference between the two diseased elbow joints on radiographs and CT images which could explain a unilateral forelimb lameness.

## Materials and methods

### Inclusion and exclusion criteria

Clinical records of the database of the Small Animal Teaching Hospital at the University of Veterinary Medicine Hannover were reviewed from February 2014 to April 2019 to identify dogs diagnosed with bilateral MCD. Dogs were eligible for participation in the study if they had a bilateral diagnosed MCD, where one forelimb presented no clinical signs and the other forelimb showed lameness and pain, swelling or crepitus during the orthopaedic examination of the elbow joint. The diagnosis was confirmed by radiographic and CT exams’. Inclusion required furthermore a complete documentation of radiographs, CTs as well as a subjective gait assessment. All the data was grouped together for each dog including name, date of birth, breed, sex, weight, age at diagnosis of MCD and hospital identification number. Cases with incomplete medical records were excluded. Exclusion criteria were concurrent elbow joint pathology such as ununited anconeal process, osteochondritis of the medial humeral condyle and flexor tendon enthesiopathy.

### Radiographs

Radiographs of each elbow joint were taken and included a mediolateral flexed and craniocaudal view. The radiographs were scored for osteoarthritis based on the IEWG guidelines. The score was modified in such a way that only the size of the osteophytes and thus the indication of arthrosis was assessed, since otherwise many radiographs images would have been assessed directly as score 2 based on our diagnosis of MCD [25] (Table 1). Incongruence wasn’t included in our modified assessment and as the dogs with concurrent elbow joint pathology such as ununited anconeal process or OCD were already excluded, these signals were irrelevant for the evaluation.

**Table 1:** Modified elbow dysplasia scoring—screening for elbow dysplasia, grading according to the IEWG Dr. H.A.W. Hazewinkel [2].

Modified Elbow Dysplasia Scoring	Radiographic Findings
0	Normal elbow joint: No evidence of sclerosis or arthrosis
1	Mild arthrosis: Presence of osteophytes < 2 mm high. Minor sclerosis of the base of the coronoid processes.
2	Moderate arthrosis: Presence of osteophytes of 2–5 mm high. Obvious sclerosis of the base of the coronoid processes
3	Severe arthrosis: Presence of osteophytes of > 5 mm high

Furthermore, Trochlear notch sclerosis (TNS) which describes a radiological term of increased bone radio-opacity in the region of the ulnar trochlear notch, was quantified. The measurements were performed as described by Draffan et al and the overall TNS ratio of sclerosis to ulnar depth was then calculated [16].

### Computer tomography

CT imaging was performed of both elbow joints from each dog with a Philips brilliance 64 slice scanner (Philips medical systems technologies LTD, Haifa Israel). Parameters varied depending on bodyweight, but most images were obtained with slice thickness of 1mm, pitch of 0.579, rotation time of 0.75 second, 120 kV and 200 mAs/slice using a bone algorithm. In order to perform the CT examination, dogs were premedicated using Acepromazin and Levomethadon, and anaesthetized using Propofol and Isoflurane in oxygen. All dogs were positioned in sternal recumbency with the front limbs extended cranially with an angle between 90° and 120° as described by Shimizu et al [19]. To avoid interference the head of each dog was pulled back. Both elbows were scanned simultaneously. Several parameters were scored as described in Table 2, as well as, the size of the fragment was measured in cm^2^. All measurements were made using a commercial imaging software (Easy Image, Denvis (CoSi dental GmbH, Sigmaringen Germany)).

**Table 2:** Computed tomographic variables studied at the medial coronoid process [21, 40, 48].

Type of pathology present at MCP	Type of fragmentation of the MCP	Fragment shape	Fragment dislocation
1. Single fragment 2. Multiple fragments 3. Fissures 4. Combination of lesions 5. None of the above lesions	1. Fragment or fissure along the radial incisure of the ulna 2. Fragmentation affecting the MCP at the apex 3. Radial incisures–Tip fragment or fissure (Combination)	1. Round 2. Pointed 3. Flattened 4. Irregular	1. Yes 2. No

### Statistical analysis

Statistical analysis were performed using the software R version 3.6.0 (2019-04-26). Following fundamental variables were recorded for the statistical calculations: A total of 42 dogs were considered. The statistical analysis was performed using 2 data sets, which contained information of the radiographs using the modified IEWG Score and TNS. Each forelimb was regarded separately so in total 84 radiographs were available for the analysis. The other data set contained information from the computed tomographic imaging, which also included 84 scans. Furthermore, data of each dog: age, breed, sex, weight, age at diagnosis and group (non-lame limb, vs. lame limb) were considered.

Descriptive statistics were generated for all variables: Metric, nearly normally distributed variables were described using the mean value (MV) and standard deviation (SD) and compared using a *t* test or Kruskal Wallis test. In contrast, skewed variables were described using the more robust median and interquartile range and checked for equal positional distribution using a nonparametric test, the Wilcoxon rank sum test or Kruskal Wallis test. Categorial variables were described using absolute (N) and relative frequencies % and compared using the χ2 independence test. A p-value of < 0.05 was considered as significant.

## Results

Forty-two dogs (84 elbow joints) with bilateral diagnosed MCD met the inclusion criteria. The most common breed were crossbreeds (10 dogs), the second most common were Labrador Retrievers (9 dogs) followed by Rottweiler (4 dogs) and Airedale Terrier (3 dogs) (Table 3). Bodyweight ranged from 10.5 to 68.5 kg (median: 33.6kg, SD: 10.5). The gender distribution in the study-population was as follows: 21 males, 9 males neutered, 8 females and 4 female neutered dogs. Age at diagnosis ranged from 7 to 104 months (median: 36.7 months, SD: 31.3).

**Table 3:** Number of breeds.

Breed	N	%
Crossbreed	10	23,8
Labrador Retriever	9	21,4
Rottweiler	4	9,5
Airedale Terrier	3	7,1
American Staffordshire Terrier	2	4,8
Bernese Mountain Dog	2	4,8
German Shepherd Dog	2	4,8
Beauceron	1	2,4
Ciobanese Mioritic	1	2,4
Elo	1	2,4
Flat Coated Retriever	1	2,4
Golden Retriever	1	2,4
Mastin de los Pirineo	1	2,4
Old English Bulldog	1	2,4
Rhodesian Ridgeback	1	2,4
Sheltie	1	2,4
Magyar Vizsla	1	2,4
Total	42	100

Depending on the gait of each dog we established two groups to examine the collected data. Group I: affected non-lame limb. Group II: affected lame limb, showing a lameness degree between a slight intermittent lameness up to a continuous non weight bearing lameness. The two groups were compared concerning the radiographic and CT differences based on this classification.

### Radiographs

Radiographs were evaluated regarding the modified IEWG score and the TNS ratio. The MV of the TNS ratio of the forelimbs from group I was 0.460 compared to the forelimbs from group II, which had an average value of 0.481. The median, as well as the 1st and 3rd quartile of the TNS ratio were also smaller in group I, but there was no significant difference (p = 0.072). Twenty five percent of the forelimbs from group I had a value less than 0.430 and 25% had a value greater than 0.498. Hence, 50% had a ratio between 0.430 and 0.498. In group II 25% of the forelimbs had a value less than 0.442 and 25% had a value greater than 0.530. Consequently 50% of the forelimbs had a TNS ratio between 0.442 and 0.530.

In the analysis of correlation, 22 forelimbs (52.4%) of the whole study population (independent of the grouping) had the same modified IEWG Score. Regarding the relative frequency of the remaining population, 16.7% of group I had a lower modified IEWG score, while group II had in 31% a higher IEWG score (see Table 4).

**Table 4:** Contingency table for variable “modified IEWG”grouped by Group I & II.

Group II	Group I				
Modified IEWG	Modified IEWG				
	0	1	2	3	Total
0	16 (38.1%)	6 (14.3%)	0 (0.0%)	0 (0.0%)	22 (52.4%)
1	6 (14.3%)	4 (9.5%)	0 (0.0%)	0 (0.0%)	10 (23.8%)
2	1 (2.4%)	1 (2.4%)	1 (2.4%)	1 (2.4%)	4 (9.5%)
3	1 (2.4%)	3 (7.1%)	1 (2.4%)	1 (2.4%)	6 (14.3%)
Total	24 (57.1%)	14 (33.3%)	2 (4.8%)	2 (4.8%)	42 (100.0%)

In comparison, forelimbs from group II had a modified IEWG score of 2 almost twice as often as those from group I, and almost three times as often a modified IEWG score of 3. The percentage distribution of the modified IEWG scores of 0 and 1 is as follows: group I: IEWG 0 = 57.1%, IEWG 1 = 33.3%; group II: IWEG 0 = 52.4%, IEWG 1 = 23.8%.

Looking at the frequency distribution of the modified IEWG score stratified by age group (4–12 months versus (vs.) > 71 months), no clear correlation could be found.

Those two age groups were applied to this study due to several different research articles and literature in which the authors outlined that especially in these two age groups the MCD is present [6–10].

### Computer tomography

To evaluate CT changes, one CT scan per limb was available for each dog, a total of 84 scans. These were evaluated according to pathology, type of fragmented coronoid process (FCP), shape of the coronoid process and dislocation (Table 2). Additionally, the size of the fragment was calculated for 82 elbow joints.

Independent of the clinical degree of lameness, 60 elbow joints (71.4%) showed a single fragment. 11 forelimbs (13.1%) had a fissure and in 10 cases (11.9%) multiple fragments were diagnosed. The type of FCP was a coronoid tip in 52.4% and 61% of the fragments were dislocated. The average size of the fragment was 0.159 cm^2^ +/- 0.129 (Table 5).

**Table 5:** General description of the lesions (CT variables).

Variable	Group I (N (%))	Group II (N (%))	p-value
**Pathology**	42 (100%)	42 (100%)	0.058
Fissure	9 (21.4)	2 (4.8)	
Combination of lesions	1 (2.4)	1 (2.4)	
Multiple Fragments	2 (4.8)	8 (19.0)	
None of the above lesions	1 (2.4)	0 (0.0)	
Single Fragment	29 (69.0)	31 (73.8)	
**Type of fragmented MCP**	42 (100%)	42 (100%)	0.019
Radial incisure–tip fragment or fissure (combination)	3 (7.1)	10 (23.8)	
Radial incisure fragment or fissure	11 (26.2)	16 (38.1)	
Tip fragment or fissure	28 (66.7)	16 (38.1)	
**Shape**	42 (100%)	42 (100%)	0.321
Flattened	11 (26.2)	14 (33.3)	
Round	13 (31.0)	12 (28.6)	
Pointed	15 (35.7)	9 (21.4)	
Irregular	3 (7.1)	7 16.7)	
**Dislocation**	42 (100%)	42 (100%)	<0.001
Yes	8 (19.9)	25 (59.5)	
No	34 (81.0)	17 (40.5)	
**Size of the Fragment**	40 (100%)	42 (100%)	0.001
Space/surface (cm^2^)	0.112 (0.106)	0.202 (0.134)	

### Pathology

Of all the dogs included (84 CT scans)—independent of the grouping—54 joints (64%) showed the same pathology in the CT scans. The following pathology occurred in Group I and Group II respectively: 24 times a single fragment, two times a fissure and once a single fragment. However, the single fragment was the most common pathology: 29/42 forelimbs from group I (69%) and 31/42 forelimbs from group II (73.8%) showed this pathology. Within the group of elbows without a lameness (group I), 21.4% (9 forelimbs) were diagnosed with a fissure on CT. Eight forelimbs (19%) of group II had multiple fragments. The remaining pathologies were rare (0–4.8%) in both groups (Table 5).

### Type of MCD

In both groups, 42.8% had the same type of MDC. In group I the tip fragment / fissure occurred most often with 66.7%, the remaining forelimbs of this group showed a radial incisure fragment or fissure in 26.2% and 7.1% of the forelimbs had a combination of radial incisure and tip fragment / fissure.

Both a radial incisure fragment or fissure, and a tip fragment or fissure were diagnosed in 38.1% of the forelimbs in group II. The remaining 23.8% had a combination of radial incisure and tip fragment or fissure (Table 5). A significant correlation (p = 0.019) was found between the different types of MCD.

### Shape

Each fragment can solely be classified either as flattened, round, pointed or irregular shape. Regarding the shape of the fragment, 26% of the forelimbs showed a coincident form in the two groups. The irregular shape occurred in group I in 7.1% of the forelimbs, and in 16.7% of the forelimbs of group II. In group I the pointed form is dominant with 35.7%, while in group II this is the second rarest shape with 21.4%. Regarding the flat and round form, the distributions between the two groups were as followed: group I: flat 26.2%; round 31%. Group II: flat: 33.3%, round: 28.6% (Table 5).

### Dislocation

Nineteen percentage of the forelimbs from group I had a dislocated fragment, compared to 59.5% of the forelimbs in group II. When considered paired, 42.9% of all elbows had a dislocation of the fragment in group II, whereas no dislocation was present in the forelimbs of group I. In 16.7% of the forelimbs there was a dislocation of the fragment in both group I and group II (Table 6). The p-value < 0.001 showed a significant difference regarding the dislocation of the fragment. One forelimb had a dislocation on the elbow which showed no lameness while the other limb showing a clinical visible lameness without a displaced fragment.

**Table 6:** Contingency table for variable “dislocation” grouped by Group I & II.

Group II	Group I		Total
	Dislocated / Yes	Not dislocated / No	
Dislocated / Yes	7 (16.7%)	18 (42.9%)	25 (59.5%)
Not dislocated / No	1 (2.4%)	16 (38.1%)	17 (40.5%)
**Total**	8 (19%)	34 (81%)	42 (100%)

### Size of the fragment

Forelimbs from group II, in regards to the median and the MV fragment sizes, had almost twice as large compared to those from group I (Group I: Median 0.09cm^2^; MV 0.11cm^2^. Group II: Median 0.16cm^2^; MV 0.20cm^2^).

The 1st quartile, 3rd quartile and maximum also took higher values in group II in comparison to group I (Tables 5 and 7, S1 Fig).

**Table 7:** Size of the fragment classified by degree of lameness: Group I + II.

Variable	Degree of lameness	N	NAs	Min	Q1	Median	Q3	Max	MV	SD	IQR
Size of the Fragment (cm^2^)	Group I	40	2	0.01	0.02	0.09	0.15	0.48	0.11	0.11	0.13
	Group II	42	0	0.01	0.12	0.16	0.28	0.60	0.20	0.13	0.16

Legend

N = number of considered values

NAs = number of missing values

Min = minimum

Q1 = first quartile

Q3 = third quartile

Max = maximum

MV = mean value

SD = standard deviation

IQR = interquartile range

Regarding the categories ‘fragment dislocation’ and ‘fragment size’ in relation the results of Table 8 and the Boxplot S2 Fig. (Supporting information) show that there is a local significance (p = 0.001). A larger fragment is more likely to dislocate than a smaller fragment.

**Table 8:** Results of the Wilcox test for fragment size grouped by dislocation.

Variable	Test statistics	95%-CI	p-value
Surface	1158	[0.049, Inf]	0.001

Legend

CI = Confidence interval

Inf = Infinity

## Discussion

This is currently the only study which analyses the disease pattern of MCD comparatively showing two different clinical pictures in one dog. Comparing to other studies, the median age of 36.7 (SD: 31.1) months of dogs at diagnosis was above the average and the median weight of 33.6kg (SD: 10.5) was similar to other studies [25, 27, 28]. Crossbreed dogs, Labrador Retrievers and male dogs were over-represented which is mirrored in other studies [25, 27, 28, 38].

The aim of the current study was to evaluate whether there are different radiographic or CT imaging findings which can explain a clinically unilateral lameness despite bilateral diagnosed MCD. The IEWG score was higher by 31% on the side of the lameness compared to the non-lame limb. However, it should be noted that both limbs had at least in 50% of cases a modified IEWG Score of 0 (57.1% vs. 52.4%). TNS values were only slightly deviated by 0.021 (0.460 group I vs. 0.481 group II), which seems to make it a worse clinical criteria to guide therapy.

CT imaging might provide a better way to differentiate the two groups. A dislocated fragment, diagnosed in 59.5% of the forelimbs showing a lameness (group II), can cause lucent defects in the subchondral bone and explain pain and lameness [39].

In this study, it could be shown that a larger fragment is more likely to dislocate than a smaller one (p = 0.001). Furthermore, there was a significant difference regarding the size of the fragment of the two groups (p = 0.001). The fragment was almost twice as large on the forelimb showing a lameness. However, a cut-off value for the size of the fragment could not be established. This study could not demonstrate an effect of the shape of the fragment, but this might be due to the measurement techniques used. It was not always simple to visualise the whole fragment properly.

A tip fragment or fissure seems to occur more often in forelimbs without a clinical visible lameness. This could be explained by the smaller size of the (fissured or fractured) fragment compared to a radial incisures, causing a smaller surface of the unstable or fractured fragment facing the joint-space. Another explanation which was shown as a significant factor by Baud et al. [40] was that the radial incisure fragment was associated to a narrower radioulnar joint space. This in turn might influence the joint mechanism negatively and cause a clinically worse lameness. It seems that this pattern was shown in the results of this study, as well shown by the p-value represented in Table 5. However, the results of group II from a stand-alone perspective showed a more or less stable distribution with regard to the type of the fragmented coronoid process, as also outlined in Table 5. Nevertheless, a study elaborated by Baud et al. [40] supports the pattern that a fissure or fragmentation of the radial incisure is more often present in dogs with a lameness that seems also present in this study since 26 forelimbs out of group II showed a radial incisure tip fragment or fissure or a combination of both which represents almost 62% of the whole population. Since only a purely clinical lameness examination was carried out and not an objective gait analysis using plate measurement for ground reaction forces, it must be questioned whether the gait analysis performed in this study was too insensitive. It is possible that the more painful limb masked the less painful limb in the clinical gait examination, so that only unilateral lameness was diagnosed by the examining veterinarians. It has already been described in the literature that dogs showed only unilateral lameness despite bilaterally diagnosed MCD and that this disease is complex and the presentation of clinical sings can be intermittent or constant [27, 41]. Concerning the clinical picture of an intermittent lameness this might be comparable to OCD, which however, has a different aetiology [42]. While the cause of OCD is a disruption of the enchondral ossification of the articular cartilage, numerous pathophysiological mechanism for MCD are postulated in the literature, finally causing a lesion of both the articular cartilage and the subchondral bone [20, 43]. This results in a more or less loose fragment which can cause—depending of the position–an intermittent lameness.

Another limitation of the study was that arthroscopy was not performed on both sides. Arthroscopy is considered the ‘‘gold standard” technique for clinical evaluation of cartilage lesions [26]. Arthroscopy can provide a good visualisation of the articular cartilage, consequently the assessment of the integrity of articular cartilage, but not the subchondral bone [39, 44]. Moreover, the detection of smaller fragments would be possible, which may be purely cartilaginous, rather than osteochondral, and therefore not detectable by CT [26]. On the other hand, the study of Morres et al [26] illustrates a significant correlation between the CT osteophyte score and the arthroscopic cartilage erosion score for the axial and the abaxial part of the MCP as well as the entire part of the MCP. This finding could question the indication for an arthroscopy. Moreover, an arthroscopic intervention may also cause progressive osteoarthritis and cartilage damage [28]. A further factor which could have been a significant parameter for the aim of this study is the radioulnar incongruence. As plain radiographs are unreliable for the detection of elbow in-congruency [45, 46], reconstructed CT scans out of sagittal and dorsal plane images should have been considered for a more accurate evaluation of the incongruence [22, 47]. As there was not always an exactly similar positioning of the dogs during the CT scans and due to the non-loaded limb nature of the CT procedure, validity of imaging might need to be questioned [15, 23]. Future studies should evaluate how loading and movement of the limb could affect CT imaging. There are already cadaver studies evaluating this effect in radiographs but they miss in CT [49, 50].

## Conclusion

In summary, a decision tree for the appropriate therapy could not be determined. Though the mentioned findings are seminal and directional parameters, which could explain the discrepancy between a clinically unaffected and a lame limb despite a radiographic MCD diagnosis. Especially the evaluation of the modified IEWG score, the dislocation and the size of the fragment should not be neglected when examining this disease pattern.

Although there are hints to what could explain partially the unequal clinical picture in the pathogenesis of MCD, understanding of the exact cause, especially for a better therapeutic approach is incomplete. More studies are urgently required to understand this complex disease pattern, following a problem-oriented therapy can be applied. Nonetheless each patient should be considered individually.

## Supporting information

S1 Fig Boxplot showing the distribution of the size of the fragments sorted by grade of lameness. (PDF)

S2 Fig Boxplot showing the statistic association between fragment size and fragment dislocation. (PDF)

S1 Data Minimal data set CT. (XLSX)

S2 Data Minimal data set radiographs. (XLSX)

S1 File Minimal data set statistic report. (PDF)
