# Supplementary material for: Comparative evaluation of radiographic and computed tomographic findings in dogs with bilateral medial coronoid disease (MCD) presenting with unilateral forelimb lameness
Source: PLoS One. 2023 Apr 10;18(4):e0282656. doi: 10.1371/journal.pone.0282656 (PMC10085011; doi:10.1371/journal.pone.0282656)

Figure 1: Boxplot showing the distribution of the size of the fragments sorted by grade of lameness

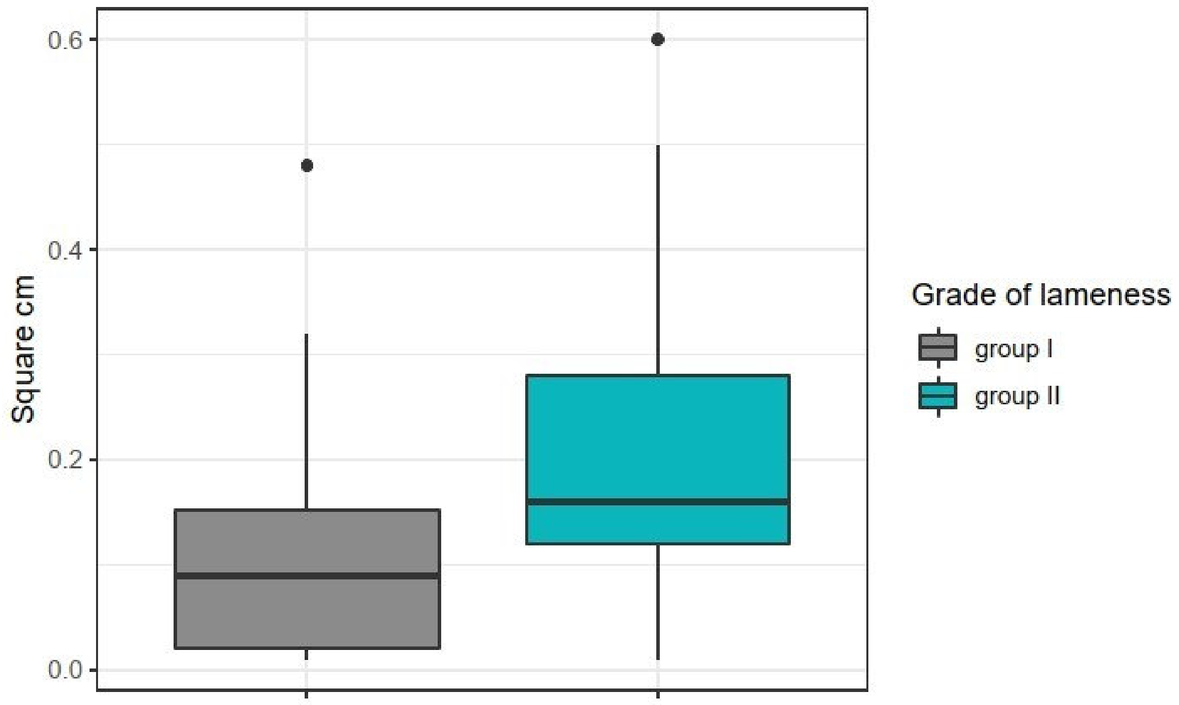

Supplement: S1 Fig — (PDF) [file pone.0282656.s001.pdf]
