# Supplementary material for: Comparative evaluation of radiographic and computed tomographic findings in dogs with bilateral medial coronoid disease (MCD) presenting with unilateral forelimb lameness
Source: PLoS One. 2023 Apr 10;18(4):e0282656. doi: 10.1371/journal.pone.0282656 (PMC10085011; doi:10.1371/journal.pone.0282656)
Supplement: S1 File — (PDF) [file pone.0282656.s005.pdf]

Chrestos
Institute

Statistical evaluation

Therapy of fragmented coronoid process in medium to large sized dogs of different breeds - Paper 2.

by Robin Brauckmann

Author
Chrestos Institute,
Chrestos Concept GmbH
(Robin Brauckmann)

Reviewer

(Sophia Seidler)

Date, SignatureDate

, Signature

©This document is the property of Chrestos and is protected by copyright. It should be treated confidentially and must not be copied or transmitted to third parties either completely or partially without the explicit permission of Chrestos.

Table of contents

1 Introduction **1**

2 Records **2**

3 Descriptor **2**
3.1 Unstratified descriptor 2
3.2 Stratified descriptor 5

4 Context analysis **8**
4.1 Observation of the variables from the X-ray images 9
4.2 Consideration of the CT variables 12

5 Conclusion **19**

6 Software information **20**

7 References **20**

List of tables

3.1 Description of the general data concerning the dogs 3
3.2 Descriptive on the general information regarding the injury (CT variables). 3
3.3 Description of the values from the X-ray images 5
3.4 Descriptor stratified to the general information regarding the injury (CT variables). by age group 6
3.5 Descriptor stratified to the general information regarding the injury (CT variables). by degree of lameness limb 7
3.6 Descriptive values from radiographs stratified by age group 8
3.7 Description to the values from the radiographs stratified by degree of lameness limb 8
4.1 Presentation of the statistical key figures of the TNS value grouped according to age group 9
4.2 Presentation of the statistical key figures of the TNS value grouped by lameness degree limb measures 9
4.3 Contingency table for the variable IEWG grouped by age class. 11
4.4 Contingency table for the variable IEWG grouped by degree of lameness limb 11
4.5 Presentation of the statistical key figures for the variable area grouped by age group 12
4.6 Presentation of the statistical key figures for the variable area grouped according to lameness degree limb 13
4.7 Contingency table for the variable pathology grouped by age class 15
4.8 Contingency table for the variable type of FPC grouped by age class. 15
4.9 Contingency table for the variable dislocation grouped by age class 15
4.10 Contingency table for the variable shape grouped by age group 16
4.11 Contingency table for the variable pathology grouped by degree of lameness limb 16
4.12 Contingency table for the variable type of FPC grouped by lameness limb . 16
4.13 Contingency table for the variable dislocation grouped by degree of lameness limb 17
4.14 P value for contingency table 4.13 17
4.15 Contingency table for the variable shape grouped by degree of lameness limb 17

List of Figures

4.1 Boxplot showing the distribution of TNS value as a function of age class (top) and limb lameness (bottom). 10
4.2 Barplot showing the frequency distribution of the IEWG value stratified by the al- ter class....... 12
4.3 Boxplot showing the distribution of the area of the fragment grouped by age class (top) and lameness grade limb (bottom). 14
4.4 Barplots showing the frequency distribution of the variable pathology (left) and type of the FPC (right) grouped by age group 18
4.5 Barplots showing the frequency distribution of the variable dislocation (left) and shape (right) grouped by age class........ 19

1 Introduction

The purpose of this analysis is to determine whether there is a difference between two groups of therapies for the treatment of fragmented coronoid process in medium-sized dogs. This is a musculoskeletal disease of the elbow joint. One elbow joint is treated with conservative therapy and the other receives surgical therapy. The elbow joint that is affected by lameness is treated surgically. The degree of lameness is between 1 and 5. conservative therapy is performed if the degree of lameness is 0.

2 Records

A total of 42 dogs are considered. The analysis is performed using two data sets. One data set contains general information regarding the dog and the disease. The dataset contains 82 observations, since each dog has two observations (one for each elbow). The other data set contains information about the radiographs. For a difference between therapies, the IEWG (International Elbow Working Group) score and the TNS (Trochelar Notch Sclerosis value) score are used. The dataset contains 82 observations. In the following, score variables are understood as the IEWG (International Elbow Working Group) score and the TNS (Trochelar Notch Sclerosis value) score. Similarly, radiographs are considered in relation to the age class of the dog. Furthermore, variables describing the disease of the elbow joint are considered descriptively and also grouped by therapy and age class are considered descriptively. Variables related to the disease of the elbow joint are also referred to as CT variables in the further course.

3 Description

The description separates into several subsections. In the first section an unstratified description is given, in the second section an unconnected stratified description is performed.

3.1 Unstratified descriptor

The following is a descriptive summary of general information about the treated dogs. In Table 3.1, metric covariates are reported using the mean (Mean), standard deviation (SD), and number of missing values. Categorical covariates are described using absolute (N) and relative frequencies (%).

Table 3.1: Description of the general data concerning the dogs

variable	N	N/A	key figures
Breed; N(P%)	42	0	
Airedale Terriers			3 (7.1%)
American Staffordshire Terriers			2 (4.8%)
Beauceron			1 (2.4%)
Bernese Mountain Dog			2 (4.8%)
Ciobanese Mioritic			1 (2.4%)
Elo			1 (2.4%)
Flat Coated Retrievers			1 (2.4%)
Golden retriever			1 (2.4%)
Labrador			9 (21.4%)
Magyar Vizsla			1 (2.4%)
Mastin de los Pirineo			1 (2.4%)
hybrid			10 (23.8%)
Old english bulldog			1 (2.4%)
Rhodesian Ridgeback			1 (2.4%)
rottweiler			4 (9.5%)
German shepherd			2 (4.8%)
sheltie			1 (2.4%)
age at diagnosis [months]; MW (SD)	42	0	36.7 (31.3)
weight [kg]; MW (SD)	42	0	33.6 (10.5)

[1] MV = mean, SD = standard deviation

In Table 3.2, the data regarding the variables related to the disease of the dog and regarding the variables related to the radiographs are given.

Table 3.2: Description of the general data concerning the injury (CT variables)

variable	N	NA	key figures
Pathology; N(P%)	84	0	
fissures			11 (13,095%)
combination of lesions			2 (2,381%)
multiple fragments			10 (11,905%)
None of the above lesions			1 (1.19%)
single fragment			60 (71,429%)
type of FPC; N(P%)	84	0	
Radial incisure – tip fragment or fissure (combined)			13 (15,476%)
Radial incisure fragment or fissure (parallel to radius)			27 (32,143%)
Tip fragment or fissure (coronoid tip)			44 (52,381%)
dislocation; N(P%)	84	0	
Yes			33 (39,286%)
No			51 (60,714%)

Shape; N (P%)	84	0	
flat			25 (29.762%)
round			25 (29.762%)
pointed			24 (28.571%)
irregular			10 (11.905%)
size of the (largest) fragments in l x b; N (P%)	82	2	
0,1 x 0,1			10 (12.195%)
0,1 x 0,6			1 (1.22%)
0,2 x 0,1			3 (3.659%)
0,2 x 0,2			3 (3.659%)
0,3 x 0,1			2 (2.439%)
0,3 x 0,2			3 (3.659%)
0,3 x 0,3			2 (2.439%)
0,3 x 0,45			1 (1.22%)
0,35 x 0,3			1 (1.22%)
0,4 x 0,2			3 (3.659%)
0,4 x 0,3			5 (6.098%)
0,4 x 0,4			3 (3.659%)
0,45 x 0,2			1 (1.22%)
0,45 x 0,3			2 (2.439%)
0,5 x 0,2			3 (3.659%)
0,5 x 0,3			6 (7.317%)
0,5 x 0,35			2 (2.439%)
0,5 x 0,4			4 (4.878%)
0,5 x 0,5			1 (1.22%)
0,55 x 0,25			1 (1.22%)
0,6 x 0,2			1 (1.22%)
0,6 x 0,25			1 (1.22%)
0,6 x 0,35			2 (2.439%)
0,6 x 0,4			4 (4.878%)
0,6 x 0,5			1 (1.22%)
0,6 x 0,6			2 (2.439%)
0,6 x 0,7			1 (1.22%)
0,7 x 0,2			1 (1.22%)
0,7 x 0,3			1 (1.22%)
0,7 x 0,4			3 (3.659%)
0,7 x 0,5			1 (1.22%)
0,7 x 0,6			1 (1.22%)
0,8 x 0,4			1 (1.22%)
0,8 x 0,5			1 (1.22%)
0,8 x 0,6			1 (1.22%)
1,0 x 0,3			1 (1.22%)
1,0 x 0,5			1 (1.22%)
1,0 x 0,6			1 (1.22%)
area [Square cm]; MW (SD)	82	2	0.159 (0.129)

[1] MW = mean, SD = standard deviation

In Table 3.3 the variables taken from the X-ray images can be seen. Table 3.3: Description of the values from the X-ray images

Variable	N	NA	Key Figures
International Elbow Working Group; N (P%)	84	0	
0			46 (54.8%)
1			24 (28.6%)
2			6 (7.1%)
3			8 (9.5%)
Trochlear Notch Sclerosis; MW (SD)	84	0	0.5 (0.1)

[1] MW = mean, SD = standard deviation

3.2 Stratified descriptor

The following tables 3.4 and 3.5, respectively, describe the above characteristics, already presented in 3.2, stratified by age group and therapy. Metric, approximately normally distributed variables are described using the mean (MW) and the standard deviation (SD) and compared using a test (t-test (stratification variable has two values, e.g. therapy) or Kruskal Wallis test (stratification variable has more than two values, e.g. age group)). Skewed variables, on the other hand, are described with the more robust median and the interquartile range (IQR) and checked for an equal position distribution using a non-parametric test (Wilcoxon rank sum test if stratification variable has two values or Kruskal Wallis test if stratification variable has more than two values). Categorical variables are described using absolute (N) and relative frequencies % and compared using the χ -independence test.[2]

Table 3.4: Descriptive information on general data regarding injury (CT variables) stratified by age group

Variable	4 to 12 months N	NAs	Key figures	At least 72 months N	NAs	Key figures	p-value
Pathology;N (%)	26	0		18	0		
Fissures			6 (23.1)			2 (11.1)	0.418
Combination of lesions			0 (0.0)			1 (5.6)	
Multiple fragments			4 (15.4)			2 (11.1)	
None of the above lesions			0 (0.0)			1 (5.6)	
Single fragment			16 (61.5)			12 (66.7)	
Type of FPC;N (%)	26	0		18	0		
Radial incisure - tip fragment or fissure (combined)			3 (11.5)			3 (16.7)	0.861
Radial incisure fragment or fissure (parallel to the radius)			7 (26.9)			4 (22.2)	
Tip fragment or fissure (coronoid tip)			16 (61.5)			11 (61.1)	
Dislocation;N (%)	26	0		18	0		
Yes			9 (34.6)			7 (38.9)	1.000
No			17 (65.4)			11 (61.1)	
Shape;N (%)	26	0		18	0		
Flat			7 (26.9)			6 (33.3)	0.902
Round			7 (26.9)			5 (27.8)	
Pointed			9 (34.6)			6 (33.3)	
Irregular			3 (11.5)			1 (5.6)	
Size of the (largest) fragment in l x b;N (%)	25	1		17	1		
0,1 x 0,1			3 (12.0)			2 (11.8)	0.483
0,1 x 0,6			0 (0.0)			1 (5.9)	
0,2 x 0,2			1 (4.0)			2 (11.8)	
0,3 x 0,1			0 (0.0)			1 (5.9)	
0,3 x 0,2			1 (4.0)			1 (5.9)	
0,3 x 0,3			0 (0.0)			1 (5.9)	
0,35 x 0,3			1 (4.0)			0 (0.0)	
0,4 x 0,2			2 (8.0)			0 (0.0)	
0,4 x 0,3			0 (0.0)			2 (11.8)	
0,4 x 0,4			1 (4.0)			1 (5.9)	
0,45 x 0,2			1 (4.0)			0 (0.0)	
0,45 x 0,3			1 (4.0)			0 (0.0)	
0,5 x 0,3			4 (16.0)			1 (5.9)	
0,5 x 0,35			1 (4.0)			0 (0.0)	
0,5 x 0,4			3 (12.0)			0 (0.0)	
0,6 x 0,2			0 (0.0)			1 (5.9)	
0,6 x 0,4			1 (4.0)			1 (5.9)	
0,6 x 0,5			1 (4.0)			0 (0.0)	
0,6 x 0,6			1 (4.0)			0 (0.0)	
0,6 x 0,7			0 (0.0)			1 (5.9)	
0,7 x 0,3			1 (4.0)			0 (0.0)	
0,7 x 0,4			1 (4.0)			1 (5.9)	
0,8 x 0,6			0 (0.0)			1 (5.9)	
1,0 x 0,6			1 (4.0)			0 (0.0)	
Area [Square cm];MW (SD)	25	1	0.164 (0.129)	17	1	0.143 (0.138)	0.619

[1] MW = mean, SD = standard deviation, p-values refer to independence test

Table 3.5: Descriptive information on general injury data (CT variables) stratified by lameness level limb

Variable	N	LH degree: 0<br>NAs	Key figures	N	LH Grade: 1-5<br>NAs	Key Figures	p-value
Pathology;N (%)	42	0		42	0		
Fissures			9 (21.4)			2 (4.8)	0.058
Combination of lesions			1 (2.4)			1 (2.4)	
Multiple fragments			2 (4.8)			8 (19.0)	
None of the above lesions			1 (2.4)			0 (0.0)	
Single fragment			29 (69.0)			31 (73.8)	
Type of FPC;N (%)	42	0		42	0		
Radial incisure - tip fragment or fissure (combined)			3 (7.1)			10 (23.8)	0.019
Radial incisure fragment or fissure (parallel to the radius)			11 (26.2)			16 (38.1)	
Tip fragment or fissure (coronoid tip)			28 (66.7)			16 (38.1)	
Dislocation;N (%)	42	0		42	0		
Yes			8 (19.0)			25 (59.5)	<0.001
No			34 (81.0)			17 (40.5)	
Shape;N (%)	42	0		42	0		
Flat			11 (26.2)			14 (33.3)	0.321
Round			13 (31.0)			12 (28.6)	
Pointed			15 (35.7)			9 (21.4)	
Irregular			3 (7.1)			7 (16.7)	
Size of (largest) fragment in l x b;N (%)	40	2		42	0		
0,1 x 0,1			9 (22.5)			1 (2.4)	0.428
0,1 x 0,6			0 (0.0)			1 (2.4)	
0,2 x 0,1			2 (5.0)			1 (2.4)	
0,2 x 0,2			2 (5.0)			1 (2.4)	
0,3 x 0,1			2 (5.0)			0 (0.0)	
0,3 x 0,2			2 (5.0)			1 (2.4)	
0,3 x 0,3			2 (5.0)			0 (0.0)	
0,3 x 0,45			0 (0.0)			1 (2.4)	
0,35 x 0,3			0 (0.0)			1 (2.4)	
0,4 x 0,2			1 (2.5)			2 (4.8)	
0,4 x 0,3			2 (5.0)			3 (7.1)	
0,4 x 0,4			1 (2.5)			2 (4.8)	
0,45 x 0,2			1 (2.5)			0 (0.0)	
0,45 x 0,3			1 (2.5)			1 (2.4)	
0,5 x 0,2			1 (2.5)			2 (4.8)	
0,5 x 0,3			3 (7.5)			3 (7.1)	
0,5 x 0,35			0 (0.0)			2 (4.8)	
0,5 x 0,4			1 (2.5)			3 (7.1)	
0,5 x 0,5			1 (2.5)			0 (0.0)	
0,55 x 0,25			1 (2.5)			0 (0.0)	
0,6 x 0,2			0 (0.0)			1 (2.4)	
0,6 x 0,25			0 (0.0)			1 (2.4)	
0,6 x 0,35			1 (2.5)			1 (2.4)	
0,6 x 0,4			2 (5.0)			2 (4.8)	
0,6 x 0,5			0 (0.0)			1 (2.4)	
0,6 x 0,6			0 (0.0)			2 (4.8)	
0,6 x 0,7			0 (0.0)			1 (2.4)	
0,7 x 0,2			1 (2.5)			0 (0.0)	
0,7 x 0,3			1 (2.5)			0 (0.0)	
0,7 x 0,4			1 (2.5)			2 (4.8)	
0,7 x 0,5			0 (0.0)			1 (2.4)	
0,7 x 0,6			0 (0.0)			1 (2.4)	
0,8 x 0,4			1 (2.5)			0 (0.0)	
0,8 x 0,5			0 (0.0)			0 (0.0)	
0,8 x 0,6			1 (2.5)			1 (2.4)	
1,0 x 0,3			0 (0.0)			1 (2.4)	
1,0 x 0,5			0 (0.0)			1 (2.4)	
1,0 x 0,6			0 (0.0)			1 (2.4)	
Area [Square cm];MW (SD)	40	2	0.112 (0.106)	42	0	0.202 (0.134)	0.001

[1] MW = mean, SD = standard deviation, p-values refer to independence test

The following Tables 3.6 and 3.7, respectively, describe the above characteristics, already presented in 3.3, stratified by age class and lameness degree of the limb. Metric, approximately normally distributed variables are described using the mean value (MW) and the standard deviation (SD) and compared using a test (t-test (stratification variable has two characteristics, e.g. degree of lameness of limb) or Kruskal Wallis test (stratification variable has more than two characteristics, e.g. age class)). Skewed variables, on the other hand, are described using the more robust median and interquartile range (IQR) and checked for an equal position distribution using a non-parametric test (Wilcoxon rank sum test if stratification variable has two values or Kruskal Wallis test if stratification variable has more than two values). Categorical variables are described using absolute (N) and relative frequencies % and compared using the χ -independence test.[2]

Table 3.6: Description of the values from the X-ray images stratified by age group

Variable	4 to 12 months			At least 72 months			
	N	NAs	Key figures	N	NAs	Key figures	p-value
International Elbow Working Group;N (%)	26	0		18	0		
0			14 (53.8)			8 (44.4)	0.748
1			7 (26.9)			7 (38.9)	
2			3 (11.5)			1 (5.6)	
3			2 (7.7)			2 (11.1)	
Trochlear Notch Sclerosis;MW (SD)	26	0	0.483 (0.054)	18	0	0.479 (0.088)	0.859

[1] MW = mean, SD = standard deviation, p-values refer to independence test

Table 3.7: Description of the values from the X-ray images stratified by degree of lameness of the limb

Variable	LH degree: 0			LH degree: 1-5			
	N	NAs	Key figures	N	NAs	Key figures	p-value
International Elbow Working Group;N (%)	42	0		42	0		
0			24 (57.1)			22 (52.4)	0.331
1			14 (33.3)			10 (23.8)	
2			2 (4.8)			4 (9.5)	
3			2 (4.8)			6 (14.3)	
Trochlear Notch Sclerosis;MW (SD)	42	0	0.460 (0.056)	42	0	0.481 (0.076)	0.159

[1] MW = mean, SD = standard deviation, p-values refer to independence test

4 Context analysis

In the first subchapter, the variables from the radiographs are considered grouped according to the age class and the degree of lameness of the limb. In the second chapter, the CT variables are grouped according to the age class and the degree of lameness of the limb.

4.1 Observation of the variables from the X-ray images

The following tables reflect the statistical ratios of the TNS value grouped by age class (Table 4.1) and by the degree of lameness of the limb (4.2). The corresponding boxplots can be seen in Figure 4.1 and 4.2.

Table 4.1: Presentation of the statistical key figures of the TNS value grouped by age group

Variable	Age group	N	NAs	Min	Q1	Median	Q3	Max	MW	SD	IQR
Trochlear Notch Sclerosis	4 to 12 months	26	0	0.390	0.440	0.470	0.535	0.600	0.483	0.054	0.095
	At least 72 months	18	0	0.310	0.432	0.470	0.535	0.650	0.479	0.088	0.103

Grade:
N: number of non-missing observations; NAs: number of missing values; Min: minimum; Q1: 1st quartile; Q3: 3rd quartile; Max: maximum; MW: mean; SD: standard deviation; IQR: interquartile range.

Table 4.2: Presentation of the statistical key figures of the TNS value grouped by degree of lameness Limb measures

Variable	Lameness degree limb	N	NAs	Min	Q1	Median	Q3	Max	MW	SD	IQR	p-value
Trochlear Notch Sclerosis	LH degree: 0	42	0	0.360	0.430	0.450	0.498	0.580	0.460	0.056	0.068	
	LH Grade: 1-5	42	0	0.310	0.442	0.470	0.530	0.680	0.481	0.076	0.088	0.072

Grade:
N: number of non-missing observations; NAs: number of missing values; Min: minimum; Q1: 1st quartile; Q3: 3rd quartile; Max: maximum; MW: mean; SD: standard deviation; IQR: interquartile range. p-value: result of paired samples t-test for metric, normally distributed variables.

The results from Table 4.2 are not significant at the local significance level of $\alpha = 5\%$. The data do not support that there is a dependence between the TNS value and the lameness level of the limb.

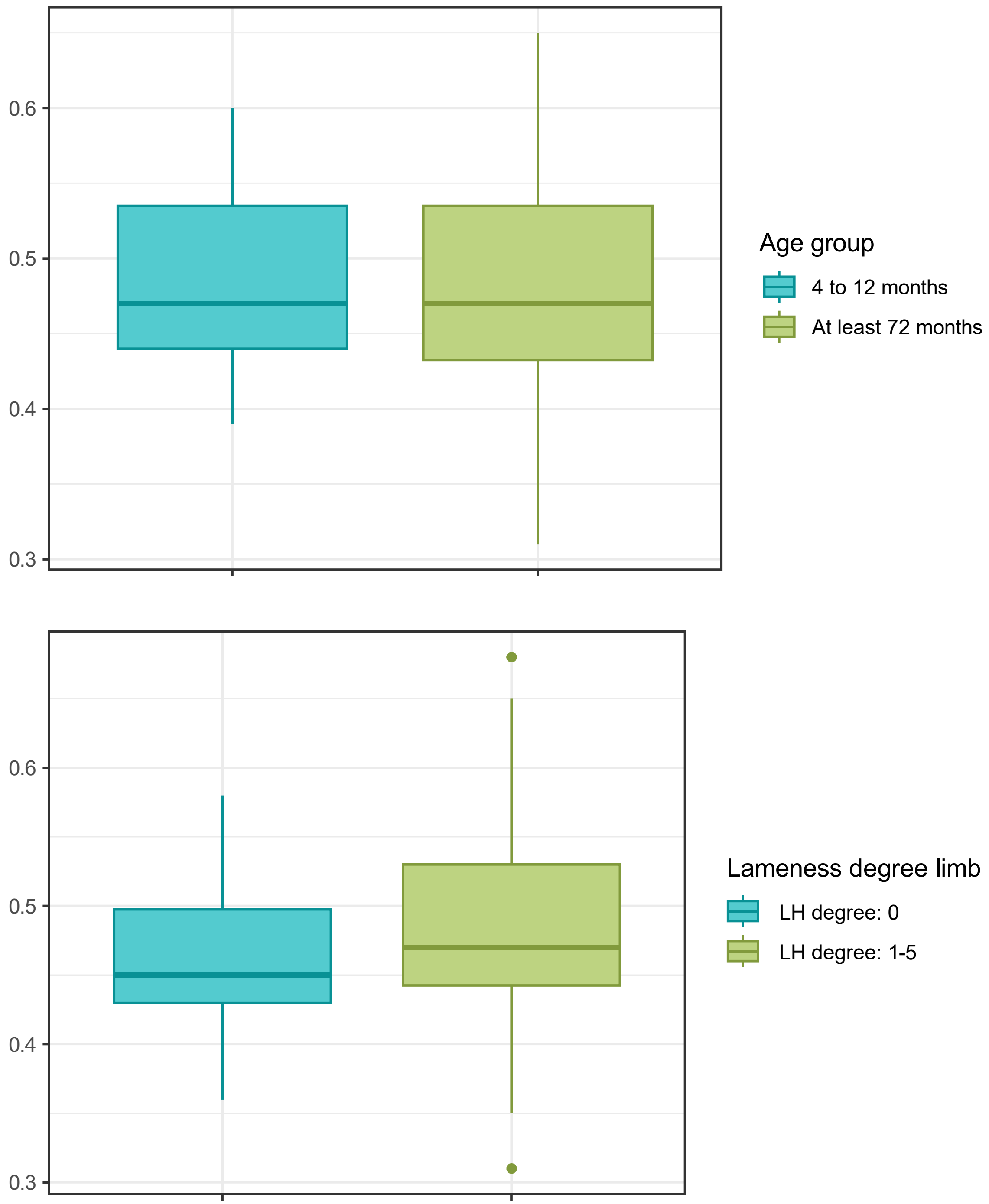

Figure 4.1: Boxplot showing the distribution of TNS value as a function of age class (top) and limb lameness (bottom).

The following tables show contingency tables in which the IEWG value is plotted together with the grouping variables age class (Table 4.3) and degree of lameness limb (Table 4.4). The plot with the grouping variable lameness degree limb is a linked sample. The percentages refer to the entire sample and not to row totals.

Table 4.3: Contingency table for the variable IEWG grouped by age class

	International Elbow Working Group				
**Age group**	**0**	**1**	**2**	**3**	**Total**
**4 to 12 months**	14 (53.8%)	7 (26.9%)	3 (11.5%)	2 (7.7%)	26 (100.0%)
**At least 72 months**	8 (44.4%)	7 (38.9%)	1 (5.6%)	2 (11.1%)	18 (100.0%)
**Total**	22 (50.0%)	14 (31.8%)	4 (9.1%)	4 (9.1%)	44 (100.0%)

Table 4.4: Contingency table for the variable IEWG grouped by degree of lameness limb

	LH degree: 0				
**LH Grade: 1-5**	**0**	**1**	**2**	**3**	**Total**
**0**	16 (38.1%)	6 (14.3%)	0 (0.0%)	0 (0.0%)	22 (52.4%)
**1**	6 (14.3%)	4 (9.5%)	0 (0.0%)	0 (0.0%)	10 (23.8%)
**2**	1 (2.4%)	1 (2.4%)	1 (2.4%)	1 (2.4%)	4 (9.5%)
**3**	1 (2.4%)	3 (7.1%)	1 (2.4%)	1 (2.4%)	6 (14.3%)
**Total**	24 (57.1%)	14 (33.3%)	2 (4.8%)	2 (4.8%)	42 (100.0%)

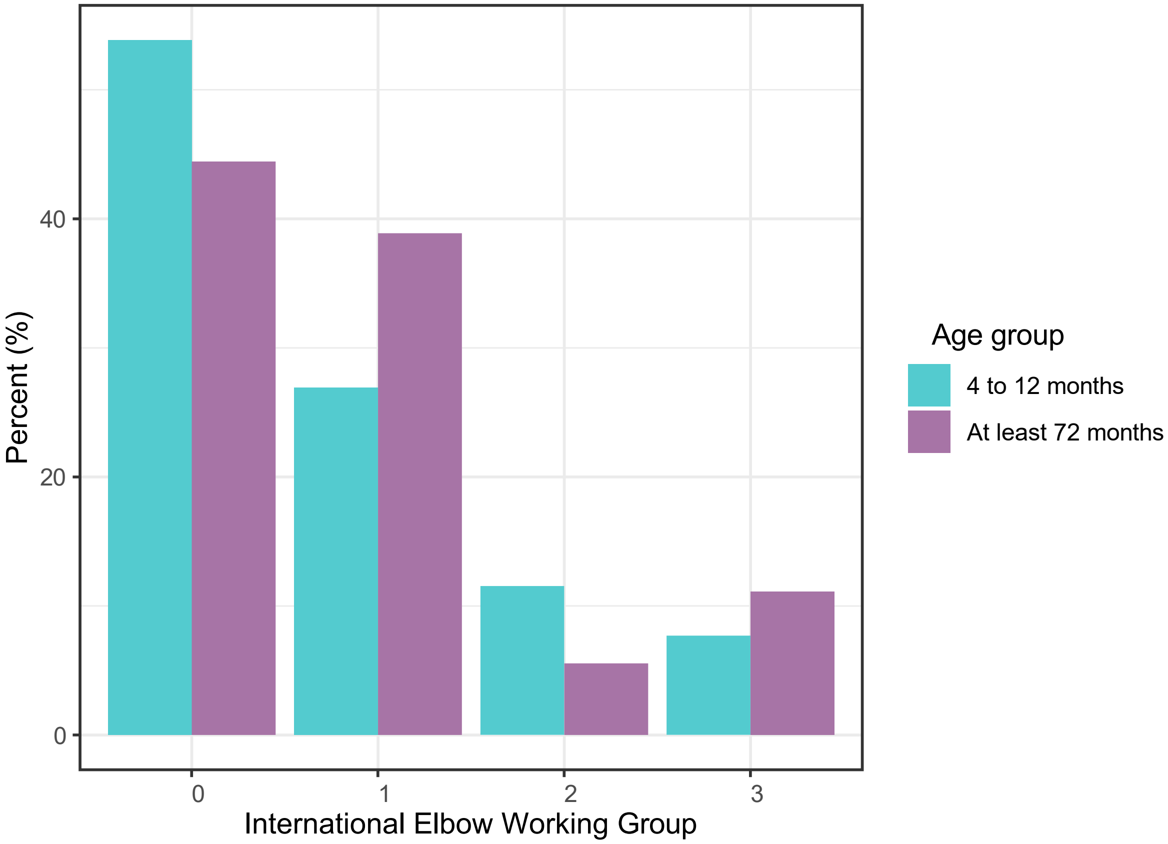

Figure 4.2: Barplot showing the frequency distribution of the IEWG value stratified by age class.

4.2 Consideration of the CT variables

For the numerical variable area, the statistical ratios are shown grouped by age class (Table 4.5) and degree of lameness limb (Table 4.6). The corresponding boxplots can be seen in the following graphs.

Table 4.5: Presentation of statistical key figures for the variable area grouped by age class

Variable	Age group	N	NAs	Min	Q1	Median	Q3	Max	MW	SD	IQR
Area	4 to 12 months	25	1	0.01	0.08	0.15	0.20	0.60	0.16	0.13	0.12
	At least 72 months	17	1	0.01	0.04	0.12	0.16	0.48	0.14	0.14	0.12

Grade:
N: number of non-missing observations; NAs: number of missing values; Min: minimum; Q1: 1st quartile; Q3: 3rd quartile; Max: maximum; MW: mean; SD: standard deviation; IQR: interquartile range.

Table 4.6: Presentation of statistical key figures for the variable area grouped by degree of lameness limb

Variable lameness degree limb		N	NAs	Min	Q1	Median	Q3	Max	MW	SD	IQR	p-value
Area	LH degree: 0	40	2	0.01	0.02	0.09	0 .15	0.48	0.11	0.11	0.13	
	LH Grade: 1-5	42	0	0.01	0.12	0.16	0 .28	0.60	0.20	0.13	0.16	0.001

Grade:
N: number of non-missing observations; NAs: number of missing values; Min: minimum; Q1: 1st quartile; Q3: 3rd quartile; Max: maximum; MW: mean; SD: standard deviation; IQR: interquartile range. p-value: result of paired t-test for metric, normally distributed variables.

The results from Table 4.6 show a significant dependence on the characteristic lameness degree limb for the characteristic area at the local significance level of $\alpha = 5\%$. The area differs in the group lameness degree 0 to the group lameness degree 1 to 5.

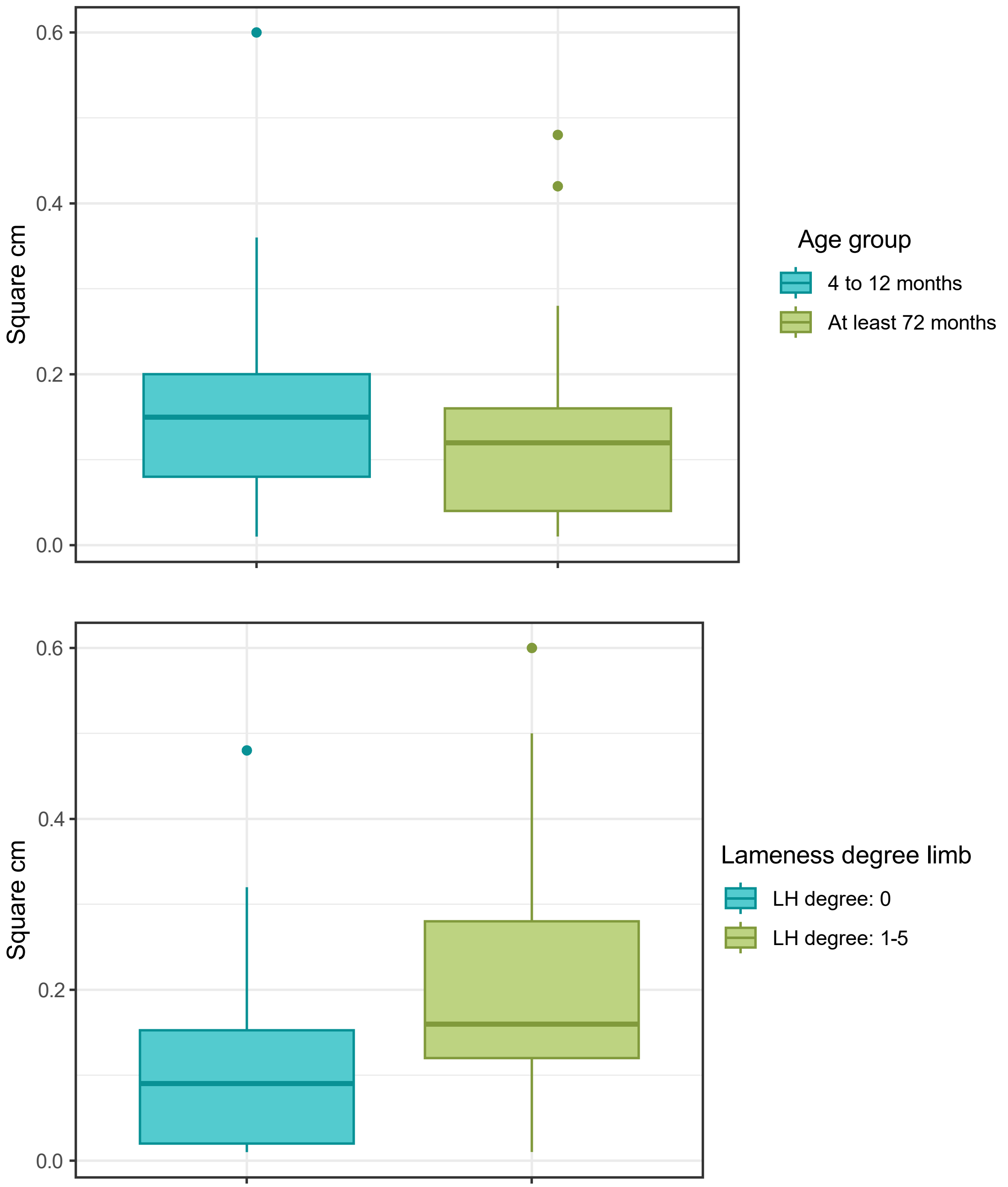

Figure 4.3: Boxplot showing the distribution of the area of the fragment grouped by age class (top) and lameness level limb (bottom).

For the categorical variables pathology, type of FPC, dislocation and shape, contingency tables with the characteristics age class and degree of lameness limb can be seen. Plots with the characteristic lameness grade limb are linked samples. The probabilities refer to all observations. In the representation with the characteristic age class, the probabilities refer to the row sums.

Table 4.7: Contingency table for the variable pathology grouped by age group

	Pathology					
Age group	1	2	3	4	5	Total
4 to 12 months	6 (23.1%)	0 (0.0%)	4 (15.4%)	0 (0.0%)	16 (61.5%)	26 (100.0%)
At least 72 months	2 (11.1%)	1 (5.6%)	2 (11.1%)	1 (5.6%)	12 (66.7%)	18 (100.0%)
Total	8 (18.2%)	1 (2.3%)	6 (13.6%)	1 (2.3%)	28 (63.6%)	44 (100.0%)

[1] 1 = fissures
[2] 2 = Combination of lesions
[3] 3 = Multiple fragments
[4] 4 = None of the above lesions
[5] 5 = Single Fragment

Table 4.8: Contingency table for the variable type of FPC grouped by age group

	Type of FPC			
Age group	1	2	3	Total
4 to 12 months	3 (11.5%)	7 (26.9%)	16 (61.5%)	26 (100.0%)
At least 72 months	3 (16.7%)	4 (22.2%)	11 (61.1%)	18 (100.0%)
Total	6 (13.6%)	11 (25.0%)	27 (61.4%)	44 (100.0%)

[1] 1 = Radial incisure - tip fragment or fissure (combined)
[2] 2 = Radial incisure fragment or fissure (parallel to the radius)
[3] 3 = Tip fragment or fissure (coronoid tip)

Table 4.9: Contingency table for the variable dislocation grouped by age group

	Dislocation		
Age group	Yes	No	Total
4 to 12 months	9 (34.6%)	17 (65.4%)	26 (100.0%)
At least 72 months	7 (38.9%)	11 (61.1%)	18 (100.0%)
Total	16 (36.4%)	28 (63.6%)	44 (100.0%)

Table 4.10: Contingency table for the variable shape grouped by age group

Age group	Form				
	flat	round	pointed	irregular	Total
4 to 12 months	7 (26.9%)	7 (26.9%)	9 (34.6%)	3 (11.5%)	26 (100.0%)
At least 72 months	6 (33.3%)	5 (27.8%)	6 (33.3%)	1 (5.6%)	18 (100.0%)
Total	13 (29.5%)	12 (27.3%)	15 (34.1%)	4 (9.1%)	44 (100.0%)

Table 4.11: Contingency table for the variable pathology grouped by degree of lameness limb

LH degree: 1-5	LH degree: 0					
	1	2	3	4	5	Total
1	2 (4.8%)	0 (0.0%)	0 (0.0%)	0 (0.0%)	0 (0.0%)	2 (4.8%)
2	0 (0.0%)	0 (0.0%)	0 (0.0%)	0 (0.0%)	1 (2.4%)	1 (2.4%)
3	2 (4.8%)	1 (2.4%)	1 (2.4%)	0 (0.0%)	4 (9.5%)	8 (19.0%)
4	0 (0.0%)	0 (0.0%)	0 (0.0%)	0 (0.0%)	0 (0.0%)	0 (0.0%)
5	5 (11.9%)	0 (0.0%)	1 (2.4%)	1 (2.4%)	24 (57.1%)	31 (73.8%)
Total	9 (21.4%)	1 (2.4%)	2 (4.8%)	1 (2.4%)	29 (69.0%)	42 (100.0%)

[1] 1 = fissures
[2] 2 = Combination of lesions
[3] 3 = Multiple fragments
[4] 4 = None of the above lesions
[5] 5 = Single Fragment

Table 4.12: Contingency table for the variable type of FPC grouped by degree of lameness limb

LH degree: 1-5	LH degree: 0			
	1	2	3	Total
1	1 (2.4%)	2 (4.8%)	7 (16.7%)	10 (23.8%)
2	2 (4.8%)	5 (11.9%)	9 (21.4%)	16 (38.1%)
3	0 (0.0%)	4 (9.5%)	12 (28.6%)	16 (38.1%)
Total	3 (7.1%)	11 (26.2%)	28 (66.7%)	42 (100.0%)

[1] 1 = Radial incisure - tip fragment or fissure (combined)
[2] 2 = Radial incisure fragment or fissure (parallel to the radius)
[3] 3 = Tip fragment or fissure (coronoid tip)

Table 4.13: Contingency table for the variable dislocation grouped by degree of lameness limb

	LH degree: 0		
**LH degree: 1-5**	**Yes**	**No**	**Total**
**Yes**	7 (16.7%)	18 (42.9%)	25 (59.5%)
**No**	1 (2.4%)	16 (38.1%)	17 (40.5%)
**Total**	8 (19.0%)	34 (81.0%)	42 (100.0%)

Table 4.14: P value for contingency table 4.13

p-value
< 0.001

The p-value refers to the performance of a McNemar test. The result of Table 4.14 shows a significant dependence on the characteristic lameness limb for the characteristic dislocation at the local significance level of $\alpha = 5\%$.

Table 4.15: Contingency table for the variable shape grouped by degree of lameness limb

	LH degree: 0				
**LH degree: 1-5**	**flat**	**round**	**pointed**	**irregular**	**Total**
**flat**	2 (4.8%)	5 (11.9%)	5 (11.9%)	2 (4.8%)	14 (33.3%)
**around**	5 (11.9%)	3 (7.1%)	3 (7.1%)	1 (2.4%)	12 (28.6%)
**pointed**	1 (2.4%)	2 (4.8%)	6 (14.3%)	0 (0.0%)	9 (21.4%)
**irregular**	3 (7.1%)	3 (7.1%)	1 (2.4%)	0 (0.0%)	7 (16.7%)
**Total**	11 (26.2%)	13 (31.0%)	15 (35.7%)	3 (7.1%)	42 (100.0%)

The following graphs present the frequency distributions of the CT variables grouped by age class.

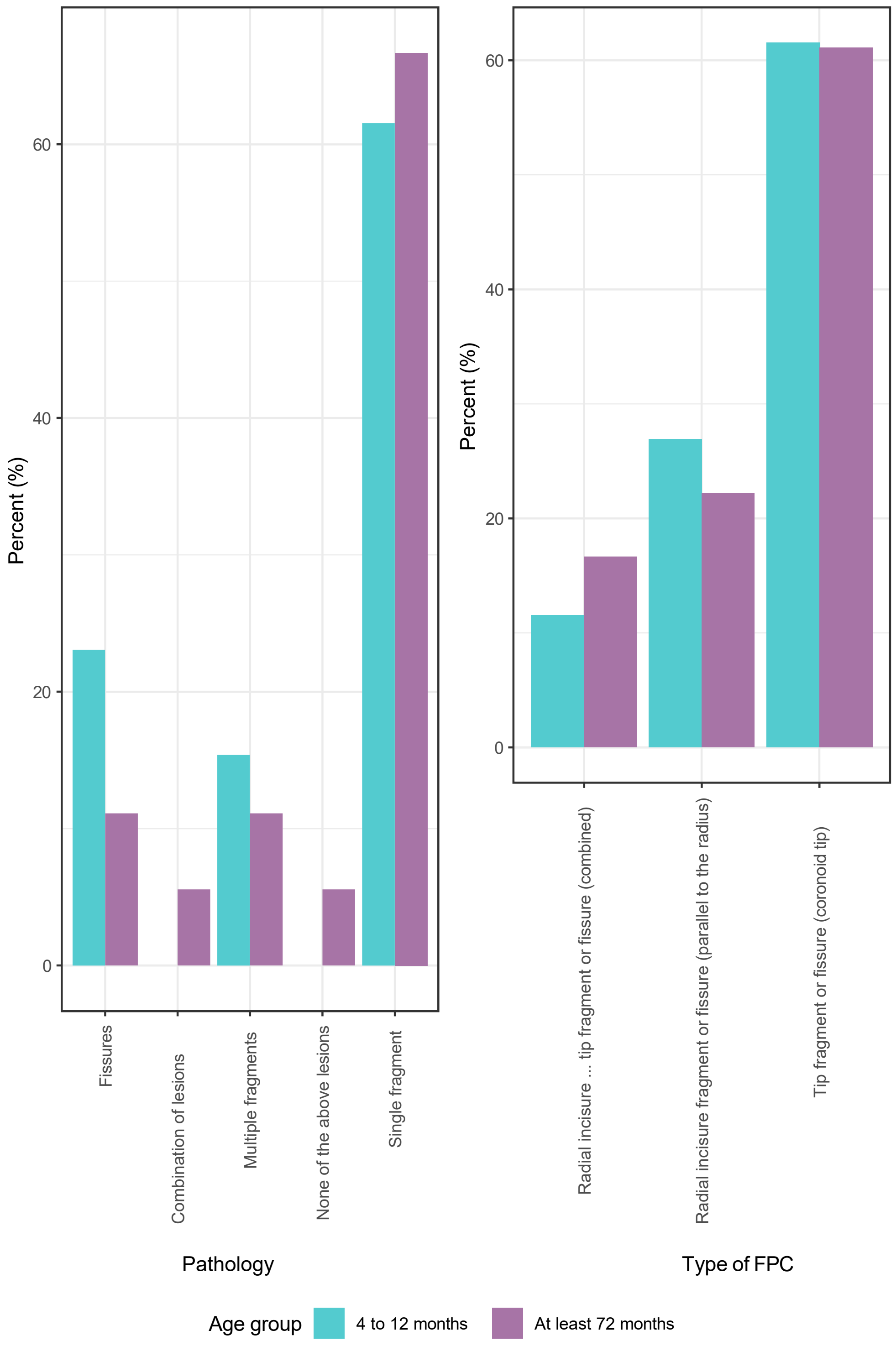

Figure 4.4: Barplots showing the frequency distribution of the variable pathology (left) and type of the FPC (right) grouped according to age group 18

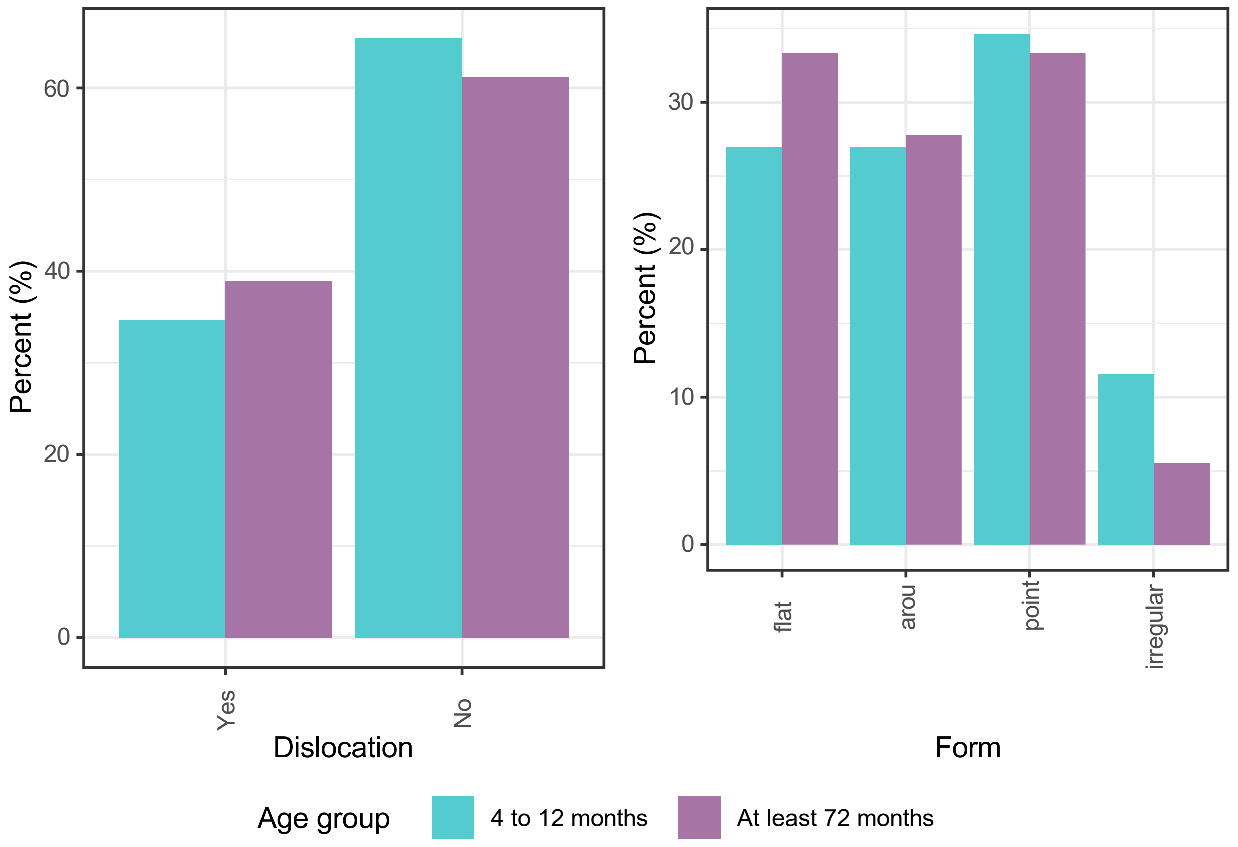

Figure 4.5: Barplots showing the frequency distribution of the variable dislocation (left) and shape (right) grouped by age class.

5 Conclusion

The 42 dogs weighed a mean of 33.6 kg (SD: 10.5) and the mean age at diagnosis was 36.7 months (31.3). A total of 17 breeds are considered; the most common breeds are the Rottweiler (4 dogs), the Labrador (9 dogs) and 10 mixed breed dogs.
From Table 3.2, it is seen that of the 84 elbow joints pathologically, 60 (71.4%) were single frag- ment and 11 (13.1%) were fissures. The type of FPC is a coronoid tip in 52.4% and 61% of the elbow joints were dislocated. The mean area of the fragment was 0.159 (SD: 0.129).
Table 3.3 shows that 54.8 % of the elbow joints have an IEWG score of 0. The mean TNS score is 0.5 (SD: 0.1).
In older dogs (at least 72 months old), 11.1 % of 18 elbow joints are pathologically fis- suric. In the younger dogs (4 to 12 months), the proportion is 23.1 % (see Table 3.4).
Table 3.5 is taken that out of 42 limbs not affected by lameness, pathologically
21.4 % are fissures. For the remaining 42 limbs affected by lameness (lameness grade 1 to 5), the rate is only 4.8 %. Differences in the type of FPC are also observed.

If the limb is affected by lameness, coronoid tips are less frequently present (38% compared to 66.7% if the limb is not affected by lameness). In limbs not affected by lameness, dislocation is present in only 19% of cases, compared to 59.5% if the limb is affected by lameness (see Table 4.13). Furthermore, the area of the fragment is almost twice as large on average when the limbs are affected by lameness (0.202 cm^2 to 0.112 cm).^{2}

In Table 3.7 and 4.2 (as well as Figure 4.1) it can be seen that the TNS value is slightly lower if the elbow joint shows the lameness grade 0. On average, the TNS value is 0.460, while the mean value for the elbow joints with lameness grade is 0.481. The median, as well as the 1st and 3rd quartiles are also smaller in the group of elbow joints with lameness grade 0.

According to Table 4.5, the area of the fragment is slightly larger if the dog is between 4 and 12 months. The median is 0.03 cm^2 larger in the group (0.15 to 0.12 cm^2) and the arithmetic mean is 0.02 ^2cm larger.

In Table 4.6 as well as Figure 4.3, it is observed that the area of the fragment is larger in the group of elbow joints affected by lameness. Minimum, 1st quartile, median, 3rd quartile, maximum and mean are larger in this group. The median in the group is 0.16, while the median in the group of elbow joints without lameness is 0.09. In the group of elbow joints without lameness degree, the 3rd quartile is only 0.15.

In Table 4.13 it is visible that only one dog has a dislocated elbow joint with lameness grade 0 and a non-dislocated elbow joint with a lameness grade between 1 and 5. However, the opposite is true in 18 dogs, so that dislocated elbow joints are present much more frequently if the elbow joints are also affected by lameness.

Table 4.14 shows that an irregular shape occurs less frequently in the elbow joints without lameness group (7.1 %) than in the group with lameness (16.7 %). In the group of elbow joints without lameness, the pointed shape is dominant (35.7 %), while in the group of elbow joints with lameness it is the second least common shape with 21.4 %.

6 Software information

R version 3.6.0 (2019-04-26)

7 References

R Core Team (2019). R: A language and environment for statistical computing. R Foundation for Statistical Computing, Vienna, Austria. URL https://www.R-project.org/.
